# A link between lipid metabolism and epithelial-mesenchymal transition provides a target for colon cancer therapy

**DOI:** 10.18632/oncotarget.5340

**Published:** 2015-10-05

**Authors:** Ruth Sánchez-Martínez, Silvia Cruz-Gil, Marta Gómez de Cedrón, Mónica Álvarez-Fernández, Teodoro Vargas, Susana Molina, Belén García, Jesús Herranz, Juan Moreno-Rubio, Guillermo Reglero, Mirna Pérez-Moreno, Jaime Feliu, Marcos Malumbres, Ana Ramírez de Molina

**Affiliations:** ^1^ Molecular Oncology and Nutritional Genomics of Cancer Group, IMDEA Food Institute, CEI UAM + CSIC, Madrid, Spain; ^2^ Cell Division and Cancer Group, Spanish National Cancer Research Centre (CNIO), Madrid, Spain; ^3^ Biostatistics Unit, IMDEA Food Institute, CEI UAM+CSIC, Madrid, Spain; ^4^ Medical Oncology, La Paz University Hospital (IdiPAZ-UAM), Madrid, Spain; ^5^ Precision Oncology Laboratory (POL), Infanta Sofía University Hospital, Madrid, Spain; ^6^ Epithelial Cell Biology Group, Spanish National Cancer Research Centre (CNIO), Madrid, Spain

**Keywords:** colorectal cancer, lipid metabolism, acyl-coA synthetases, stearoyl-CoA desaturase, epithelial-mesenchymal transition

## Abstract

The alterations in carbohydrate metabolism that fuel tumor growth have been extensively studied. However, other metabolic pathways involved in malignant progression, demand further understanding. Here we describe a metabolic acyl-CoA synthetase/stearoyl-CoA desaturase ACSL/SCD network causing an epithelial-mesenchymal transition (EMT) program that promotes migration and invasion of colon cancer cells. The mesenchymal phenotype produced upon overexpression of these enzymes is reverted through reactivation of AMPK signaling. Furthermore, this network expression correlates with poorer clinical outcome of stage-II colon cancer patients. Finally, combined treatment with chemical inhibitors of ACSL/SCD selectively decreases cancer cell viability without reducing normal cells viability. Thus, ACSL/SCD network stimulates colon cancer progression through conferring increased energetic capacity and invasive and migratory properties to cancer cells, and might represent a new therapeutic opportunity for colon cancer treatment.

## INTRODUCTION

Colorectal cancer (CRC) is one of the most deathly tumors worldwide. In addition to genetic modifications, factors causing metabolic alterations such as obesity, sedentarism and westernized diet, are risk factors for the disease [1]. Metabolic reprogramming is a distinguished cancer hallmark [2]. It is well known the Warburg effect by which cancer cells preferentially drive glucose metabolism to lactate production under aerobic conditions [3]. In addition to carbohydrate metabolism, other metabolic pathways have been found to be altered in cancer [4]. Lipid metabolism, represents an extremely relevant source of energy and structural and biosynthetic resources [5, 6].

De novo fatty acid synthesis is required for membrane production, essential for cell growth and proliferation. In addition, an appropriate ratio between saturated and monounsaturated fatty acids (SFA and MUFA, respectively) is needed for proper membrane fluidity and cell function. An increased content of MUFA has been observed in several cancers [7] and it has been proposed as a predictive marker [8]. The rate-limiting enzyme converting SFA into MUFA is steraroyl-CoA desaturase-1 (SCD), which introduces a double bound into palmitic acid and stearic acid giving rise to palmitoleic and oleic acid, respectively [9]. SCD have been described upregulated in several types of human tumors and its expression has been correlated with malignant transformation, proliferation and survival [10–14].

Acyl-CoA synthetases (ACSL) catalyze the conversion of long chain fatty acids to acyl-CoA, which is critical for phospholipid and triglyceride synthesis, lipid modification of proteins as well as for fatty acid β-oxidation [15]. These enzymes have been related to carcinogenesis [16, 17]. Although involved in the same reaction, enzymes of ACSL family differ in substrate specificity, subcellular localization, and tissue distribution. In fact, these enzymes use saturated and unsaturated fatty acids of 8–22 carbons as substrates with the exception of ACSL4 that shows a clear preference for arachidonic acid as a substrate [6]. ACSL4 increases proliferation, tumor growth and survival in breast, prostate, colon and liver malignancies [18–21]. Remarkably, ACSL4 overexpression channels fatty acids preferentially towards phosphatidylinositol, an effect not observed for ACSL1 [22]. ACSL1 has been recently involved in lipid metabolism alterations in cancer [23], including in a screening in colon cancer of lipid metabolism-related genes recently conducted in our research group [24, 25].

Epithelial-mesenchymal transition (EMT) is a conserved morphogenetic program characterized by the loss of epithelial phenotype and the gain of mesenchymal features [26]. Epithelial cells discard their cell polarity and cell-cell adhesion and acquire a mesenchymal morphology with migration and invasion capability. These properties promote metastasis and the development of several neoplasias [27]. Loss of E-Cadherin and nuclear translocation of β-Catenin constitute known EMT biomarkers. β-Catenin translocation to the nucleus leads to invasion genes transcription [28]. This requires avoiding β-Catenin rapid cytosolic degradation by the proteasome, achieved upon GSK3β phosphorylation [29]. An implication of carbohydrates metabolism in the EMT phenotype acquisition and maintenance has been reported [30, 31] although no connection has been yet described with fatty acid metabolism.

Here we identify a cooperative metabolic network comprising ACSL1, ACSL4 and SCD, involved in CRC progression. The simultaneous overexpression of ACSL1, ACSL4 and SCD induces EMT, increases cellular migration and invasion and stimulates several survival pathways. Besides, the combination of low doses of pharmacological inhibitors for ACSL and SCD, dramatically decreases cancer cell viability in a synergistic manner. Moreover, such combination also reduces cell viability of chemotherapy resistant colon cancer cells without having any effect in normal colonocytes. Finally, the clinical relevance of these findings is underscored in CRC patients with tumors displaying ACSL1/ACSL4/SCD simultaneous overexpression that show an increased risk of relapse compared to other patients within the same clinicopathological stage.

## RESULTS

### Lipid metabolism enzymes overexpression confers EMT properties to colon cancer cells

**Figure 1:**
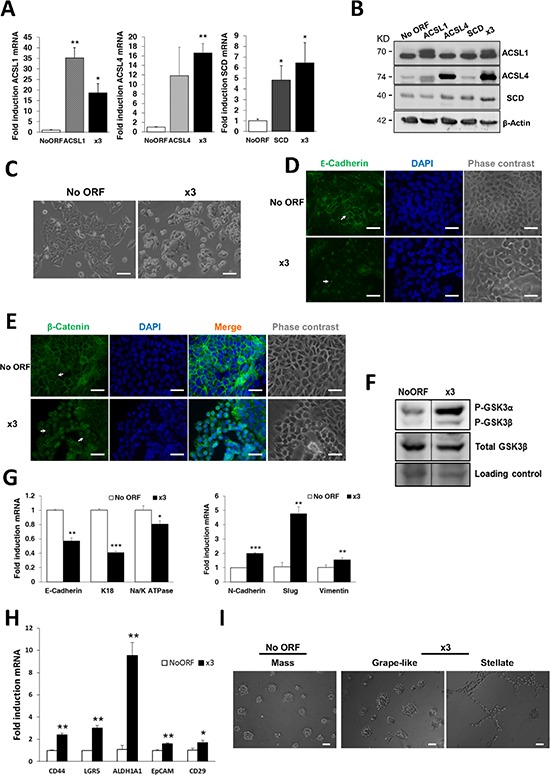
ACSL1, ACSL4 and SCD overexpression induces EMT in CRC cells **A.** Stable cell lines overexpressing ACSL1, ACSL4 and SCD, alone or in combination (x3) were generated using lentiviral transduction and expression levels of every gene were measured by RT-QPCR. **B.** Protein expression levels of ACSL1(upper band), ACSL4 and SCD for each cell line were detected by Western Blot with specific antibodies. β-Actin detection was used as a loading control. **C.** Representative phase contrast images showing atypical morphology of x3 cells compared to control No ORF cells. Scale bars, 100 μm. **D–E.** Representative immunofluorescence images of E-Cadherin (green) (D) or β-Catenin (green) (E) cellular distribution of No ORF and x3 cells. Arrows indicate the different expression patterns in the cellular distribution of E-Cadherin (D) or β-Catenin (E) in both types of cells. Nuclei were stained with DAPI (blue) and equivalent phase contrast images were taken. Scale bars, 50 μm. **F.** Levels of GSK3 phosphorylation detected by Western Blot using a phospho-specific antibody (Ser21/9). Total GSK3β levels detection and Ponceau-stained band served as loading controls. Bottom panel: Quantification of bands intensity showing the ratio of phosphorylated to total protein. **G.** RT-QPCR analysis of epithelial (*E-Cadherin, K18* and *Na+/K+ATPase β1*) and mesenchymal genes (*N-Cadherin, Slug* and *Vimentin*) for x3 cells compared to levels in No ORF control cells. **H.** The expression levels of the colon cancer stem cell markers *CD44, Lgr5, ALDH1A1, EpCAM* and *CD29* were measured by RT-QPCR in x3 cells and compared to the levels in No ORF control cells. **I.** Phase contrast pictures showing the different morphology of spheroids formed by No ORF and x3 cells cultured under 3D on-top assay conditions. Scale bars, 100 μm. Experiments in A, G and H were performed in triplicates (*n* = 3). Results represent the mean ±SD (*n* = 3). *, *p* < 0.05, **, *p* < 0.01, ***, *p* < 0.001.

The aim of this study was to perform a comparative analysis of the potential involvement of ACSL1 and ACSL4 in CRC, in addition to analyzing whether their combination with the related enzyme SCD might increase tumorigenesis due to the probable role of SCD in preventing lipotoxic effects that could result from ACSL overexpression. For this purpose we generated human colon cancer stable cell lines overexpressing either ACSL1, ACSL4 or SCD (ACSL1 cells, ACSL4 cells, and SCD cells, respectively) or the three genes simultaneously (x3 cells), transducing the DLD-1 CRC cell line with specific lentiviruses. An equivalent control vector which does not express any ORF was also used to generate the No ORF cell line. Transcript levels of ACSL1, ACSL4 and SCD were measured to ensure that stable expression of the constructs was achieved (Figure 1A), which was further confirmed by Western Blot analysis of the proteins levels (Figure 1B).

Unlike No ORF controls, nor any of the cell lines overexpressing any of these genes individually, x3 cells displayed a more spindle-like shape with a more scattered distribution, resembling mesenchymal or fibroblast-like phenotype (Figure 1C). Since this morphological change resembled an EMT phenotype characterized by loss of cell-cell adhesion, we next analyzed the expression of the epithelial marker E-Cadherin. As shown in Figure 1D, the membrane-associated pattern of expression of E-Cadherin was disrupted upon ACSL1, ACSL4 and SCD simultaneous overexpression. Mislocalization of E-cadherin was more evident in the areas where the more fusiform and rounded x3 cells were present (Figure 1D, bottom panel, arrow). Moreover, loss of β-Catenin from the membrane and a clear increase in nuclear localization was also found in x3 cells when compared with No ORF control cells (Figure 1E). This is also in agreement with a loss of epithelial characteristics and gain of an EMT phenotype, since its translocation to the nucleus would lead to the transcription of invasion genes [28]. Figure 1F shows how GSK3β inhibitory phosphorylation is highly increased in x3 cells, allowing β-Catenin nuclear translocation. β-Catenin acts as a transcriptional coactivator at the nucleus promoting the transcription of EMT genes [32]. Accordingly, together with a decrease in the expression of the epithelial markers *E-Cadherin, K18* and *Na^+^/K^+^ATPase β1*, x3 cells increased the expression levels of EMT associated genes *N-cadherin, Slug* and *Vimentin* (Figure 1G) which are normally not expressed in the markedly epithelial DLD-1 cells.

Accordingly with the lack of any morphological change, no mislocalization of E-cadherin nor changes in epithelial markers were observed in cell lines singly overexpressing any of these genes (Supplementary Figure S1A–S1B). Interestingly, an increase in GSK3β phosphorylation was also observed in SCD cells (Supplementary Figure S1C). In contrast, only cells overexpressing ACSL1, but not ACSL4 or SCD (data not shown) displayed an up-regulation of *N-cadherin* and *Slug* expression (Supplementary Figure S1D). These results suggest that each gene might be contributing in different aspects of EMT, though the cooperation of the three genes is needed to trigger the EMT program.

Cells undergoing EMT have been described to present cancer stem cells features [33]. Accordingly, x3 cells were significantly enriched in the well-established markers of CRC stem cells *CD44, LGR5, ALDH1A1, EpCAM* and *CD29* when compared with No ORF cells (Figure 1H). Moreover, x3 cells form tridimensional colonies with differential morphologies when grown in matrigel. While No ORF cells displayed the normal DLD-1 spheroid round morphology termed as “mass” [34, 35] (Figure 1I, left panel), x3 cells whether presented “grape-like” spheroids with loose cell-cell contacts (Figure 1I, central panel) or even “stellate” colonies with invasive projections able to bridge several cell colonies (Figure 1I, right panel). This again highlights the more mesenchymal behavior of x3 cells and suggests an invasive capacity for these cells.

### ACSL/SCD metabolic network fuels migration, invasion and cell survival

**Figure 2:**
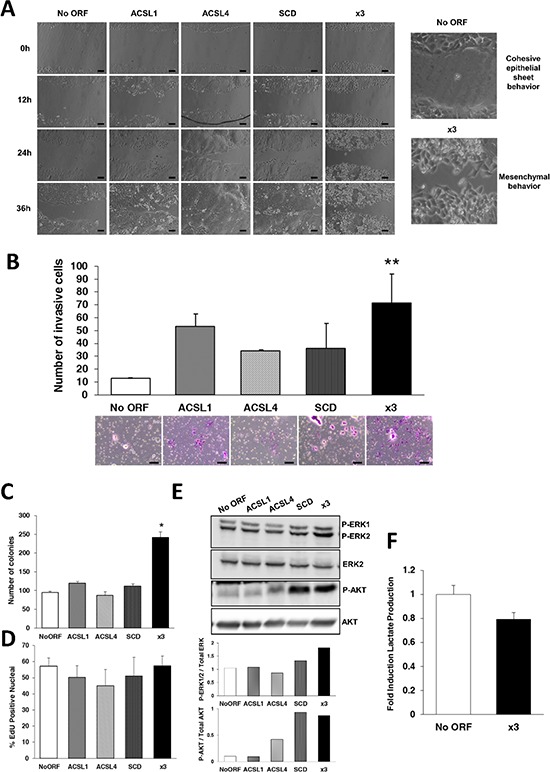
Combination of ACSL with SCD overexpression stimulates migration, invasion and colony formation without major effects on proliferation **A.** Phase contrast pictures of wound healing assay comparing migratory capacities of No ORF, ACSL1, ACSL4, SCD and x3 cells. Magnification (right panels) shows the different behavior of control and x3 cells at 36 hours of wound closure. Scale bars, 100 μm. **B.** Boyden chamber transwell assay of invasion through Matrigel. After 48 hours, cells were fixed and stained with crystal violet (bottom panels) and counted under an optical microscope. Migratory cells were quantified as the average number of cells found in five random microscope fields in three independent inserts. Scale bars, 50 μm. **C.** Clonogenic assay quantification. Individual cells were plated and colonies formed were grown during 2 weeks. Data are presented as the average number of colonies resultant for each cell line. **D.** Quantification using fluorescence microscopy of EdU incorporation as a measure of cell proliferation. **E.** Erk1/2 and Akt phosphorylation were detected by Western Blot with phospho-specific antibodies (Tyr204 Erk and Thr308 Akt). Total Erk and Akt were used as their respective controls. Bottom panels: Quantification of bands intensity showing the ratio of phosphorylated to total protein. **F.** Quantification of extracellular L- Lactate produced by No ORF and x3 cells. Experiments in B, C, D and F were performed in triplicates (*n* = 3). Results represent the mean ±SD (*n* = 3). *, *p* < 0.05, **, *p* < 0.01.

The acquisition of migratory and invasive properties is a general feature of cells undergoing EMT, crucial for metastasis formation and cancer progression. In order to check if the combination of ACSL and SCD overexpression could confer cancer cells a gain of migratory capacity, we performed wound healing assays. Figure 2A shows how x3 cells present an increased migration ability compared to No ORF cells. As illustrated in the magnification, x3 cells close the wound upon random migration, characteristic of a mesenchymal behavior. In contrast, No ORF control cells, ACSL1, SCD, and more markedly ACSL4, display the collective unidirectional migration of cohesive epithelial sheets. Furthermore, poorly invasive DLD-1 cells gain the ability to invade through Matrigel (Figure 2B) upon ACSL1, ACSL4 and SCD simultaneous overexpression (x3). The invasion capacity was also increased upon individual overexpression, especially in the case of ACSL1 cells. However, any of these individual effects was less prominent that the one observed in x3 cells. These results highlight the cooperative effect in promoting migration and invasion achieved when these three metabolic genes act in a combined manner.

Cancer cells often use metabolic strategies to promote cell survival and proliferation. To analyze whether an increased lipid metabolism caused by ACSL1, ACSL4 and SCD overexpression could be implicated in these processes, we first performed clonogenic assays to monitor long-term growth and survival. Figure 2C shows that x3 cells present more than twice efficiency in colony formation compared to control cells. In contrast, none of the individual overexpression caused this increase in the number of colonies formed indicating again that it is the combination of the three genes that confers the cells the more aggressive characteristics.

To directly assay cell proliferation we analyzed the incorporation of EdU as a measure of DNA synthesis rate. No significant changes in proliferation were found in any of the stable cell lines (Figure 2D). Since colony formation needs cell proliferation, we also performed those EdU assays in conditions of confluence to make these assays more comparable to the growth conditions in the colonies produced during clonogenic assays. Again, we do not find substantial differences in proliferation in any of the cell lines (Supplementary Figure S2). A number of EMT regulators have been reported not to increase proliferation, and invasive cells associated to decreased cell cycle progression [36, 37]. We next analyzed Erk and Akt activation, two well-known pathways for cell survival involved in EMT induction mainly through GSK3β regulation [38]. We found a significant activation of both cascades in x3 cells (Figure 2E). Besides, a clear increase in Erk and Akt phosphorylation was found in SCD cells, as well as in ACSL4 cells in the case of Akt phosphorylation. These partial activations per se may be insufficient but may contribute to the full effect and phenotype observed in x3 cells.

All these data suggest that unlike other tumor metabolic pathways such as the Warburg effect, the ACSL/SCD network acts specifically promoting invasive and pro-survival properties without major effects on cell proliferation. In agreement with that, x3 cells did not show increased glycolytic activity when compared with No ORF control cells (Figure 2F). This reveals that EMT promotion might require particular metabolic advantages other than pro-proliferative aerobic glycolysis.

### AMPK signaling counteracts the EMT phenotype triggered by the ACSL/SCD signaling network

**Figure 3:**
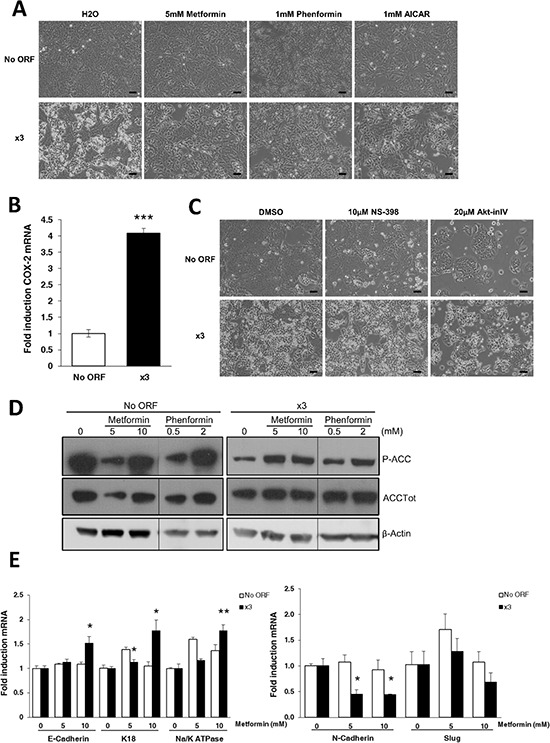
AMPK activation rescues normal DLD-1 epithelial phenotype **A.** Representative phase contrast pictures of No ORF and x3 cells treated either with 5 mM metformin, 1 mM phenformin, 1 mM AICAR or vehicle (water) for 48 hours. Scale bars, 100 μm. **B.** COX-2 expression levels of x3 cells compared to control No ORF cells, measured by RT-QPCR. **C.** Pictures represent cells treated for 48 hours either with the COX-2 inhibitor NS-398 (10 μM), the Akt inhibitor IV (Akt-inIV, 2 μM), or with an equivalent amount of DMSO as control. Scale bars, 100 μm. **D.** Western blotting of ACC phosphorylation upon 48 hours of metformin (5 and 10 mM) or phenformin (0.5 and 2 mM) treatment in No ORF and x3 cells. Total protein extracts were blotted using a phospho-specific antibody (Ser79) and a total ACC antibody. β-Actin detection was used as a loading control. **E.** RT-QPCR analysis shows mRNA levels of epithelial markers (*E-Cadherin, K18* and *Na+/K+ATPase β1*) upon 48 hours of metformin treatment (5 and 10 mM) in No ORF and x3 cells (left panel). Similarly, right panel presents the levels of the mesenchymal markers *N-Cadherin* and *Slug* in these cell lines under similar treatments. The values for every gene are presented as fold change referenced to their respective control (0 mM metformin) within each cell line. Results in C and E were performed in triplicates, and represent the mean ±SD. *, *p* < 0.05, **, *p* < 0.01, ***, *p* < 0.001.

In addition to classical oncogenic pathways [38], nutrient sensing and inflammation regulators have been more recently added to the list of EMT-inducers [39, 40]. Since ACSL/SCD overexpression may represent an energetic advantage, we wondered if the master regulator of energy balance, AMPK, was involved. This kinase, considered to act as a tumor suppressor by inhibiting tumor metabolism and associated cell growth signaling pathways [41] has been described to suppress EMT by regulating the Akt-MDM2-Foxo3 signaling axis [42]. Accordingly, treatment with three different AMPK activators; metformin, phenformin and AICAR, was able to rescue the DLD-1 epithelial phenotype (Figure 3A).

Besides sharing arachidonic acid as a substrate, ACSL4 regulates COX-2 expression and thus prostaglandin production [19], also implicated in cell malignancy and EMT promotion [43]. Since x3 cells, but not any of individually overexpressing cell lines, show increased levels of COX-2 expression (Figure 3B); we surmised that x3 EMT might be mediated by a COX-2 dependent mechanism. Conversely, treatment with the COX-2 inhibitor NS-398 had no effect on the x3 cells phenotype reversion (Figure 3C, central panel). Moreover, despite the strong Akt activation in x3 cells (Figure 2E), the treatment with Akt inhibitor IV (Figure 3C, right panel) was also inefficient to revert x3 mesenchymal features. This drug only inhibited cell viability of both cell lines without altering their morphological features. These data pointed towards AMPK signaling as key for maintaining the DLD-1 cells epithelial phenotype. This was further demonstrated by analysis of the downstream AMPK target, Acetyl-CoA Carboxylase (ACC), which is inactivated upon phosphorylation at Ser79 (P-ACC). The ACC inhibitory phosphorylation was induced upon metformin or phenformin treatment in x3 cells (Figure 3D). Furthermore, metformin treatment stimulated the expression of the epithelial markers *E-Cadherin, K18* and *Na^+^/K^+^ATPase β1* and decreased the expression levels of the EMT-induced genes *N-Cadherin* and *Slug* in x3 cells without increasing those levels in control No ORF cells (Figure 3E).

### Stage-II CRC patients with simultaneous overexpression of ACSL1, ACSL4 and SCD have worse clinical outcome

**Figure 4:**
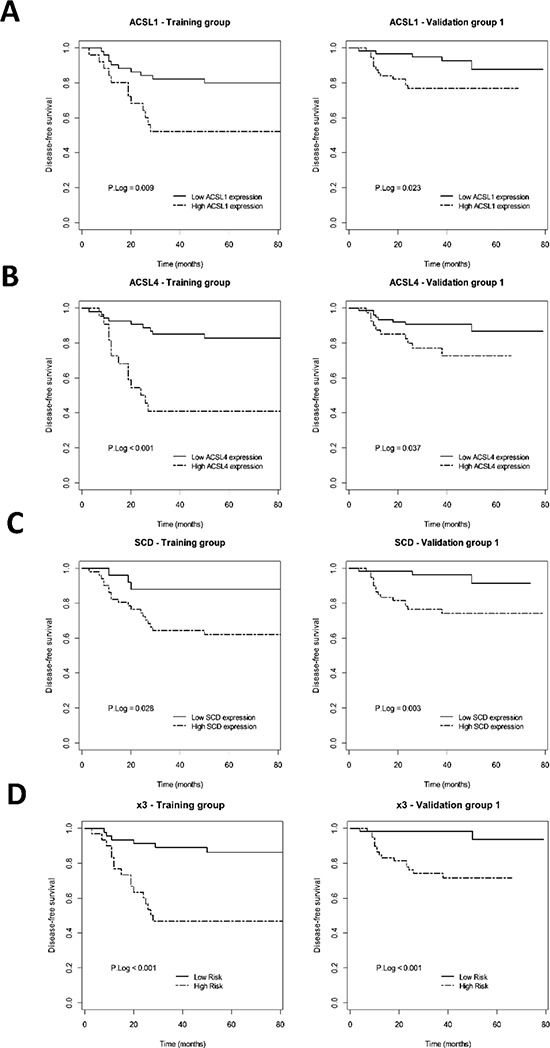
Prognostic value of ACSL1/ACSL4/SCD individual or simultaneous (x3) overexpression in early-stage CRC patients Kaplan-Meier plots representing DFS for ACSL1 **A.** ACSL4 **B.** SCD **C.** and x3 **D.** overexpressing early-stage CRC patients and p Log Rank value in the training and validation groups are shown.

In order to determine the clinical relevance of ACSL/SCD network activation in CRC, we analyzed the putative association between the simultaneous overexpression of ACSL1, ACLS4 and SCD with clinical outcome in a set of 77 samples from stage-II CRC patients. Towards this aim we have recently developed a global analysis of lipid metabolism-related genes [25]. Median follow-up of these patients was 71.5 months. The 3-year disease free survival (DFS) was 72.3%, 22 patients (28.57%) relapsed. Clinico-histopathological characteristics of these patients are detailed in Supplementary Table S1. An initial screening performed showed that 16 out of 70 lipid metabolism-related genes analyzed, including ACSL1, ACLS4 and SCD, might have a putative association with tumor aggressiveness [25]. Thus, we performed a comparative analysis of the relationship between the individual expression of these genes and their combined overexpression in these patients. As expected from the screening, Kaplan-Meier plots for disease free-survival of ACLS1, ACSL4 or SCD showed an association between high expression of each of these genes and poorer clinical outcome (Figure 4A, 4B and 4C). Moreover, the multivariable model x3 (combined expression of ACSL1, ACSL4 and SCD) showed that the simultaneous overexpression of the ACSL/SCD network resulted in a stronger and more potent association with patient relapse (Figure 4D and Table 1), confirming the acquisition of increased aggressive properties of CRC tumors with these characteristics.

**Table 1:** Univariate and Multivariate Cox regression analyses for Disease-free survival of the ACSL1, ACSL4 and SCD genes, the multivariable model x3 (combined expression of ACSL1, ACSL4 and SCD) in stage II CRC samples from the training group and the validation group

Variable	Training group (*n* = 77)								Validation group (*n* = 119)							
	Low Risk		High Risk		Unadjusted		Adjusted#		Low Risk		High Risk		Unadjusted		Adjusted#	
	R	N	R	N	HR (95% CI)	*P*	HR (95% CI)	*P*	R	N	R	N	HR (95% CI)	*P*	HR (95% CI)	*P*
**ACSL1**	10	51	12	26	2.93 (1.26–6.81)	0.013	2.34 (0.91–6.02)	0.082	5	61	13	58	3.12 (1.11–8.76)	0.021	3.86 (1.16–12.79)	0.017
**ACSL4**	9	54	13	23	4.92 (2.09–11.62)	0	5.6 (2.19–14.32)	0	8	78	10	41	2.59 (1.02–6.57)	0.045	2.31 (0.85–6.27)	0.1
**SCD**	3	26	19	51	3.57 (1.06–12.08)	0.018	3.13 (0.9–10.93)	0.046	3	57	15	62	5.32 (1.54–18.38)	0.002	4.08 (1.12–14.9)	0.017
**x3**	6	46	16	31	5.36 (2.09–13.74)	0.000	4.99 (1.81–13.77)	0.001	2	58	16	61	9 (2.07–39.21)	0.0002	8.68 (1.9–39.66)	6E-04

Abbreviations: HR (95% CI), hazard ratio and corresponding 95% confidence interval from adjusted or unadjusted Cox regression analyses; P, *p* value from adjusted or unadjusted Cox regression analyses; N, N° of patients in each risk group; R, N° of patients with relapse.

# Cox regression analyses were adjusted for T stage, Vascular invassion, Perineural invassion, Bowel Obstruction/Perforation and Age > 70.

We validated these results in an independent and bigger cohort of 119 stage II CRC patients with a median follow-up of 43 months and 3-year DFS of 86.1% respectively, from which 18 patients relapsed (15.3%) (Clinico-pathological characteristics summarized in Supplementary Table S1). The univariate cox regression analysis in this validation group also showed a statistical association between overexpression of each gene and clinical outcome (Figure 4, Table 1). The multivariate analysis in this validation group further demonstrated that the combined overexpression of these genes was associated with a higher increased risk of relapse of stage-II CRC patients, with higher power and statistical strengthening than any of these genes individually (x3: HR (95% CI) = 8.68 (1.9–39.66); *p* = 6e-04), which is more than 2-fold higher than any of these genes separately, results that were confirmed after adjusting for clinical confusing factors (Table 1).

These results, not only show the clinical relevance of the overexpression of these genes in CRC, but also demonstrate that their combination is associated with an increased aggressiveness of CRC tumors in a clinical setting. Collectively, these results imply that ACSL1, ACSL4 and SCD increased expression might act as a marker of poor prognosis which contributes to reduced disease free survival of colon cancer patients. Very likely, the EMT program triggered by the ACSL1/ACSL4/SCD axis would be implicated in facilitating the spread of the disease.

### Combined pharmacological inhibition of the ACSL/SCD network synergy to selectively inhibit cancer cells viability and mesenchymal features

**Figure 5:**
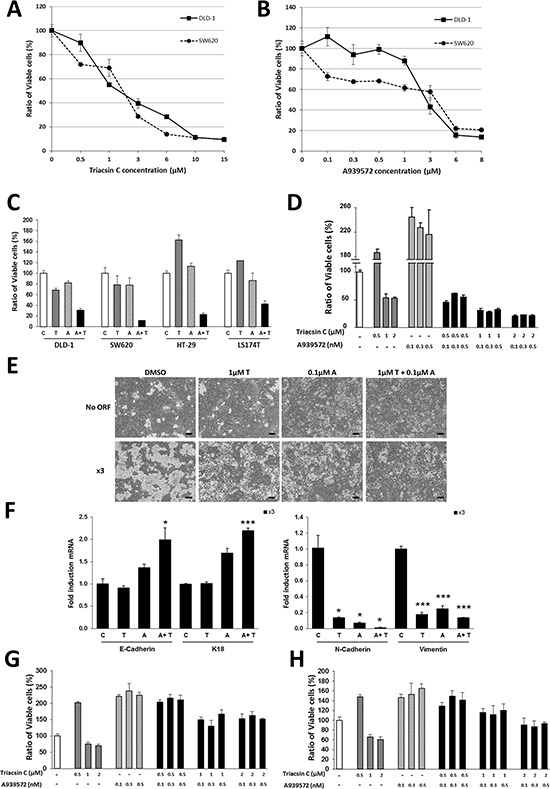
Synergistic effect of ACSL and SCD inhibitors on CRC cells **A–B.** Dose–response curves of the MTT cell viability assays after 48 hours treatment of SW620 and DLD-1 colon cancer cells with increasing concentrations of ACSL inhibitor Triacsin C (A) or SCD inhibitor A939572 (B). **C.** MTT cell viability assays in DLD-1, SW620, HT-29 and LS174T colon cancer cell lines show synergistic action on cell viability of low doses of Triacsin C (1 μM) and A939572 (0.5 μM) upon 48 hours treatment. **D.** Response of SW620–5FU-R cells in MTT assays to the 48 hours treatment with different concentrations of Triacsin C and/or A939572. **E.** Representative phase contrast pictures of No ORF and x3 cells treated with vehicle (DMSO), Triacsin C (1 μM) and/or A939572 (0.1 μM) for 48 hours. Scale bars, 100 μm. **F.** RT-QPCR analysis showing the x3 cells mRNA levels of the epithelial markers *E-Cadherin, K18* and *Na+/K+ATPase β1* and the mesenchymal markers *N-Cadherin* and *Slug* upon 48 hours of treatment with Triacsin C (1 μM) and/or A939572 (0.1 μM). **G–H.** MTT cell viability assays in normal colonocytes. CCD18Co (G), or CCD841 cells (H), were treated for 48 hours with different concentrations of Triacsin C and/or A939572. Data are represented as the mean ±SD (*n* = 3) in all the plots. C = Vehicle, T = Triacsin C, A = A939572, A+T = A939572+Triacsin C. *, *p* < 0.05, ***, *p* < 0.001.

The robust protumor action of ACSL1/ACSL4/SCD network, together with the fact that all three of them are lipid metabolism enzymes, makes these druggable proteins attractive targets for cancer therapy. For this reason, we evaluated the combined effect on colon cancer cell viability of Triacsin C, a specific inhibitor of ACSL, and the SCD inhibitor A939572, both previously reported to reduce tumor growth both *in vitro* and *in vivo* [14, 17]. Triacsin C was able to decrease cell viability in DLD-1 and SW620 cell lines in a dose-dependent manner (Figure 5A). A similar effect was achieved upon treatment with A939572 (Figure 5B). Since the use of high concentrations of pharmacological inhibitors may cause side effects and act on other pathways, the combination of the two compounds might inhibit ACSL/SCD network and exert a similar action using much lower concentrations of both compounds.

Lower concentrations of Triacsin C and A939572 alone or in combination were used in a panel of colon cancer cell lines (Figure 5C). As expected, treatment with 1 μM Triacsin C or with 0.5 μM A939572 moderately decreased DLD-1 and SW620 cells viability, but importantly, the combined action of both inhibitors caused a strong cancer cell viability reduction. Furthermore, Triacsin C and A939572 individually had no effect on the viability of the more resistant HT-29 and LS174T cells; however, a potent inhibition was observed when those low doses of both compounds were used (Figure 5C).

In addition, to analyze the effect of these compounds on CRC cells resistant to conventional chemotherapy, we used a subclone of SW620 cells previously described [44], which is resistant to 5-Fluorouracil. The drugs had little (Triacsin C), or no effect (A939572) on SW620–5FU-R when applied separately. Nonetheless, a strong cooperative effect was found upon simultaneous treatment with ACSL and SCD inhibitors (Figure 5D).

Since the EMT program observed in x3 cells is triggered by the combined action of ACSL with SCD, we wanted to assay the effect on EMT of their inhibitors. Combined treatment with low doses of these drugs was able to reverse the mesenchymal phenotype of x3 cells to a situation resembling No ORF cells (Figure 5E). Furthermore, this was accompanied by an increase in the expression of epithelial markers and a reduced expression of EMT-genes in x3 cells (Figure 5F). Hence, the pharmacological inhibition of ACSL/SCD network is able to counteract the x3 cells EMT features.

Finally, the ability of a compound to discriminate cancer cells from their normal counterparts is the basis for successful cancer treatment. For this reason, we assayed the effect of Triacsin C and A939572 on the viability of normal colonocytes. Very importantly, the same inhibitors concentrations that caused a massive loss of viability of colon cancer cell lines (Figure 5C and 5D) were completely ineffective when applied to normal colonocytes CCD18Co (Figure 5G). This was further confirmed using another normal colon cell line, CCD841 (Figure 5H) resulting in a similar behavior. Thus, the inhibition of ACSL1/ACSL4/SCD axis by means of the selective effect of these drugs on cancer cells arises as a promising therapeutic strategy.

## DISCUSSION

We show for the first time how the overexpression of lipid metabolism genes can lead to EMT induction and increased migratory and invasive properties. ACSL1/ACSL4/SCD overexpression in CRC cells causes a phenotypic switch to a mesenchymal-like condition characterized by rounded cell morphology and E-Cadherin and β-Catenin mislocalization (Figure 1D and 1E). This phenotypic change, which leads to an increased migration and invasion (Figure 2) is only achieved upon overexpression of the three proteins, presumably having each of them more partial but differential functions. ACSL1 may be an initiator of the process since its overexpression stimulates EMT genes expression (Supplementary Figure S1D). ACSL1 seems also to be fundamental for invasive abilities (Figure 2B) which could be caused by an increased expression of invasive genes such as matrix degrading enzymes. For its part, SCD overexpression strongly increases inhibitory phosphorylation of GSK3β (Supplementary Figure S1C). This may be a consequence of SCD overexpression-induced Akt activation (Figure 2E) which phosphorylates and inactivates GSK3β [38, 45, 46], avoiding β-catenin degradation and allowing further EMT signaling. Indeed, SCD knock-down impairs β-catenin signaling and inhibits EMT-like behavior of metastatic breast cancer cells [47]. SCD may maintain the network signaling through Erk and Akt stimulation (Figure 2E) which are key for EMT programs [48]. In fact, oleic acid, the main product of SCD, activates Akt pathway while palmitic acid has the opposite effect [49]. Very likely, SCD maintenance of the cancer preferred MUFA/SFA ratio, is the driving force of Erk and Akt signaling as observed in lung and prostate cancers [10, 50]. Hence, the SCD-induced increase in MUFA content seems crucial to maintain EMT and tumor dissemination. We found no evidence highlighting any specific role of ACSL4 overexpression in EMT onset. However, due to its substrate preference for arachidonic acid, and the reported crosstalk with COX-2 [18, 19], ACSL4 could favor prostaglandin production and other inflammatory mediators of EMT [40, 43]. Conversely, ACSL4-overexpressing cells display a more epithelial-like behavior in wound healing assays (Figure 2A). It could be possible that upon feeding EMT, ACSL4 could provide the necessary plasticity to revert to the epithelial phenotype through a mesenchymal–epithelial transition (MET) [26], crucial for the growth and establishment of metastasis.

Even though Warburg-like metabolism seems to be present in most malignancies, alternative energy sources can be used depending on the tumor context [4], suggesting an intrinsic metabolic flexibility for cancer cells. ACSL/SCD network promotes migration and invasion without main effects on cell proliferation (Figure 2). Hence, it is reasonable to think that an enhanced lipid metabolism has more implications other than support proliferation like pro-proliferative aerobic glycolysis. In fact, x3 cells show no increase, but a slight decrease in lactate production (Figure 2F). Altered lipid metabolism may be more directed to provide EMT and invasion. Interestingly, it has been reported a monoacylglycerol lipase-governed fatty acid network which drives invasion and cancer pathogenesis [51]. This could be initially driven by an EMT mechanism as the one generated by the ACSL1/ACSL4/SCD axis. On the other hand, lipids are important signaling molecules triggering a number of protumor pathways. SCD inhibition decreases the synthesis of PIP_3_, crucial for Pi3K/Akt signaling [10]. Furthermore, arachidonic acid, the main ACSL4 substrate, is one of the most common fatty acids of PIP_3_. ACSL, especially ACSL4, also stimulate fatty acid entry into β-oxidation [15] which generates not only ATP but also redox power necessary to counteract tumor oxidative stress [5]. Lipid anabolism and catabolism alterations have been proposed to coexist in tumor cells [52]. Therefore, an increased fatty acid catabolism caused by ACSL overexpression, along with increased de novo fatty acid synthesis and advantageous MUFA content upon SCD upregulation could generate an array of protumorigenic signals capable of supporting malignancies through an initial EMT program.

The abnormal metabolism of x3 cells provides an energetic advantage crucial to EMT promotion and invasiveness. Such is the case, that if energy homeostasis is disrupted either upon AMPK signaling reactivation (Figure 3) or upon simultaneous chemical inhibition of ACSL and SCD (Figure 5E and 5F), epithelial features are rescued. Defective AMPK signaling seems to be the main mechanism to drive EMT by the ACSL/SCD network since interference with other pathways already described to cause EMT such as Pi3K/Akt or COX-2 [43, 48] had no effect on the original phenotype recovery (Figure 3C). AMPK could be suppressing EMT through the regulation of the Akt-MDM2-Foxo3 signaling axis as recently proposed [42]. This is in agreement with the increased Akt signaling observed in x3 cells (Figure 2E). However, Akt inhibitor IV fails to revert EMT suggesting additional pathways affected by AMPK signaling that are also needed for a complete rescue. On the other hand, SCD downregulation activates AMPK [10, 53] but no link has been established between AMPK and ACSL. Since free fatty acids are able to activate AMPK [54] ACSL overexpression could prevent this activation through the conversion of fatty acids into Acyl-CoAs.

ACSL1 implication in triglyceride synthesis [55] together with the function of ACSL4 in phospholipids channeling [22] suggests an increased lipid synthesis upon ACSL overexpression. Its associated lipotoxicity would be counteracted by SCD overexpression. Accordingly, x3 cells have 20% less fat content than No ORF cells (data not shown). Thus, either a more efficient energetic use of lipids or an abnormally increased metabolism allows malignant features of x3 cells. Their advantage does not rely on exogenous lipid supply as no variations were found when experiments were performed upon serum lipid depletion or serum content reduction (data not shown). Besides, there was no increase in lipid incorporation neither of oleic acid nor palmitic acid into lipid vesicles upon ACSL1, ACSL4 or SCD overexpression (data not shown). In agreement with that, the described monoacylglycerol-lipase invasive signature also shows independence from extrinsic fatty acids supply [51].

**Figure 6:**
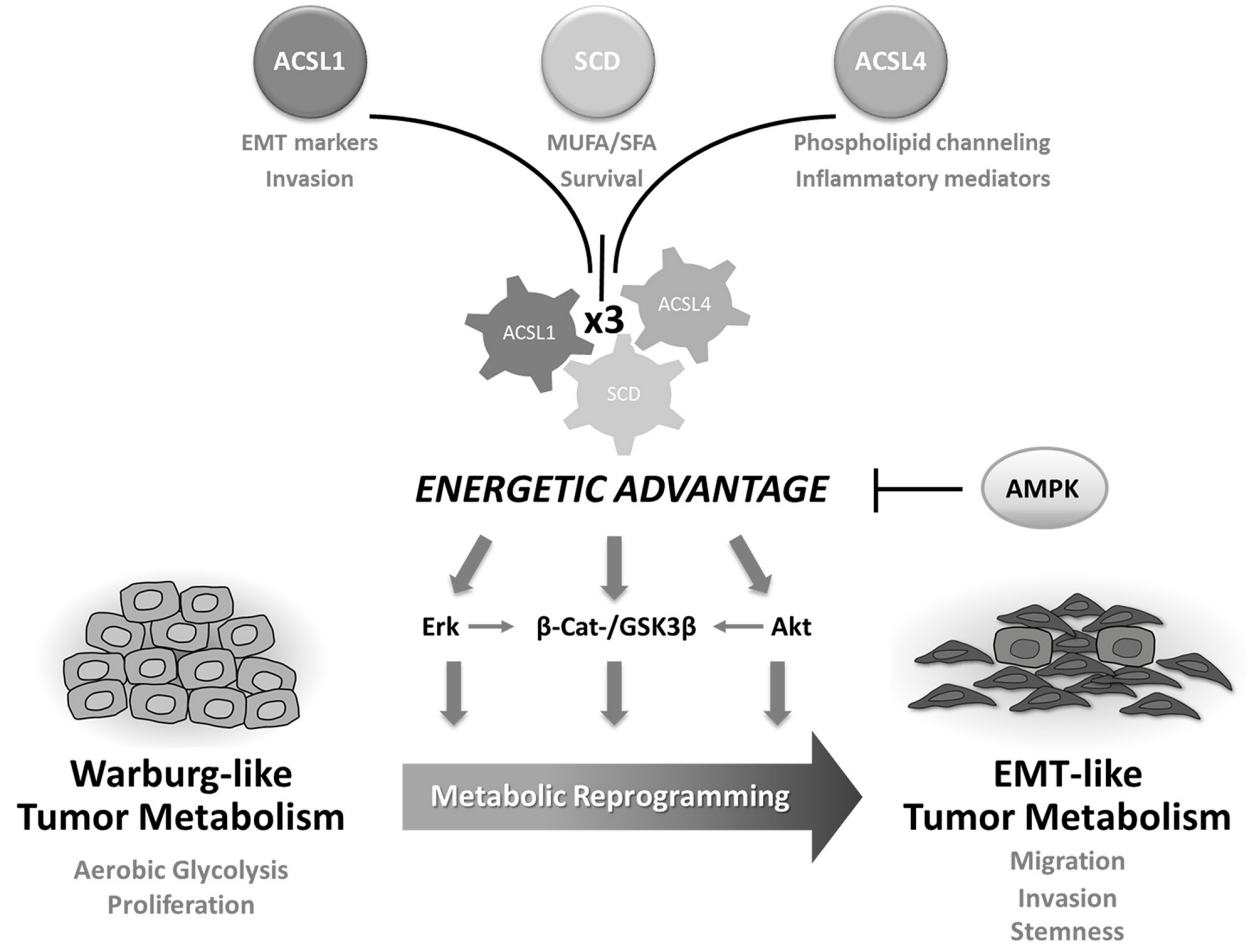
ACSL/SCD-mediated tumor metabolic reprogramming The combined action of ACSL1, ACSL4 and SCD confers an energetic advantage to tumor cells that stimulates several EMT-promoting and survival pathways. Lipid metabolism switches Warburg-like tumor metabolism into EMT-like tumor metabolism leading to a gain of mesenchymal and stem cell properties as well as migratory and invasive capabilities. This process can be reversed upon re-activation of AMPK signaling.

The combined overexpression of ACSL1/ACSL4/SCD shows a poorer outcome and higher risk of relapse when compared to the upregulation of each of the genes separately in stage II-CRC patients (Figure 4 and Table 1). This suggests an increased aggressiveness of tumors overexpressing ACSL/SCD network. This fatty acid metabolism switch that acts through an EMT program is thus an example of how different types of metabolic reprogramming can be used by tumors to increase their malignity depending on the needs and the environment (Figure 6). The combination of ACSL/SCD inhibitors synergistically reduces cancer cells viability without decreasing normal cells viability (Figure 5C, 5G and 5H). Furthermore, this is also effective in CRC cells resistant to conventional chemotherapy (Figure 5D). Therefore, this sort of “synthetic lethality” caused by the combined action of ACSL and SCD inhibitors represents a new way of addressing tumor metabolism and may designate the ACSL/SCD axis interference as a promising opportunity for cancer treatment.

## MATERIALS AND METHODS

### Cell culture

Colon cancer cell lines, normal colon cell lines, and HEK-293T cells, were obtained from American Type Culture Collection (ATCC, Manassas, VA, USA), cultured in DMEM supplemented with 10% FBS and maintained under standard conditions. SW620–5FU-R cells were generated by exposing parental cells to increasing concentrations of the drug for 15 months as previously described [44]. All cell lines were authenticated by microsatellite genotyping. The 3D on top assays were performed as previously described [56]. Cells were plated in Matrigel (BD Biosciences, Franklin Lakes, NJ. USA) at a density of 10000 cells per well in 24-well plates and cultured for up to a week. Images were captured using a Leica DM IL microscope (Leica Microsystems, Wetzlar, Germany), with a 10X Plan Fluotar objective and registered using Leica Application Suite (LAS, Leica).

### Reagents

Commercial antibodies used are listed in Supplementary Table S2. Antibody against SCD-1 [57] was a kind gift from Dr. Jean-Baptiste Demoulin, Université Catholique de Louvain, Brussels, Belgium. Anti-human ACSL4 was generously provided by Dr. Stephen Prescott, University of Utah, Salt Lake, USA and Dr. Diana Stafforini, Huntsman Cancer Institute, University of Utah, USA, and used as indicated in [58]. Triacsin C was purchased from Santa Cruz (Santa Cruz, CA, USA); A939572 was from Biofine International (Biofine International Inc, Vancouver, Canada); Metformin, Phenformin, AICAR, NS-398 and Akt Inhibitor IV were from Sigma-Aldrich, (Sigma-Aldrich, St. Louis, MO, USA).

### Lentivirus-mediated stable overexpression of ACSL1, ACSL4 and SCD

HEK 293T cells were transfected using Lipofectamine 2000 (Life Technologies, Carlsbad CA, USA) with lentiviral vectors expressing ACSL1, ACSL4, SCD or No ORF empty vector (DNA 2.0, Menlo Park CA, USA) along with a set of packaging plasmids (Addgene, Cambridge MA, USA). DLD-1 cells were infected with supernatant produced upon 48 hours transfection in HEK293T cells and 4 μg/μl polybrene (Millipore) as coadjutant. Selection was performed during 1 week by adding 2 μg/ml puromycin, 3 μg/ml blasticidin, and 150 μg/ml hygromycin, (Sigma) for SCD, ACSL4, and ACSL1 constructs respectively.

### Western blot

Cells were lysed in Laemmli buffer, proteins were separated by SDS–polyacrylamide gel electrophoresis and transferred onto a nitrocellulose membrane (Bio-Rad Laboratories, Hercules, CA, USA). The membranes were blocked using 5% nonfat dry milk in TBS 0.05% Tween-20. Primary antibodies were incubated overnight at 4°C and upon 1 hour incubation with secondary antibodies signal detection was performed using the Clarity Western ECL Substrate (Bio-Rad). β-actin determination or Ponceau stain were used as loading controls.

### Cell viability assay

Cell viability was determined by seeding 25000 cells per well in 24-well plates and treated with the corresponding compounds for 48 hours. Upon treatment, cells were incubated 3 hours with 3-(4,5-dimethyl-thyazol-2-yl)-2,5-diphenyl-tetrazolium (MTT, Sigma). The resultant formazan was dissolved in DMSO and its quantity determined by measuring absorbance at 560 nm. Data were expressed as the ratio of viable cells (%) which represents the percentage of viable cells upon treatment compared with the non-treated cells (100% ratio of viable cells).

### L-Lactate quantification

Cells were seeded at a density of 5000 cells per well in a 96-well plate. At 12 hours, medium was changed to 2% FBS and kept for 24 hours at 37°C before quantification. Using Cayman's Glycolysis cell-based assay (Cayman, Ann Arbor, MI, USA, 600450) extracellular L-Lactate was measured by determining absorbance at 490 nm.

### Quantitative real-time PCR

Total RNA was extracted using Tri Reagent (Sigma). 400 ng of RNA were reverse-transcribed using the High Capacity RNA-to-cDNA Master Mix system (Life Technologies). qPCR was performed in the 7900HT Real-Time PCR System (Life Technologies) using VeriQuest SYBR Green qPCR Master Mix (Affymetrix, Santa Clara, CA, USA) and gene specific primers listed in Supplementary Table S3. Values were corrected by *GAPDH* expression. The 2^−ΔΔCt^ method was applied to calculate the relative gene expression.

### Immunofluorescence

Cells were fixed with 4% paraformaldehyde for 10 minutes at room temperature, then permeabilized 20 minutes with 0.5% triton X-100, and stained with the primary antibody (1:100) followed with incubation with Alexa 488-conjugated anti-mouse secondary antibody (1:1000) and/or with DAPI (Prolong Gold antifade, Life Technologies) to visualize nuclei. Images were captured using a Leica DM IL microscope, with a 20X Plan Fluotar objective and registered using Leica Application Suite (LAS).

### EdU incorporation assay

Cells were plated on coverslips and incubated 24 hours before treatment with 10 μM EdU for 3 hours. In the case of the EdU assays in confluent conditions, EdU treatment was added once cells reached confluence. Cells were fixed with paraformaldehyde (4%) for 8 minutes and incorporation of EdU was assayed using Click-iT® EdU Alexa Fluor® 488 Imaging Kit (Life Technologies). Coverslips were mounted on slides and nuclear DNA stained with DAPI. Images were captured using a Leica DM IL microscope, with a 40X Plan Fluotar objective and registered using LAS software.

### Invasion assays

A density of 50000 cells was seeded into the inserts of a BD Matrigel^™^ invasion chamber (BD Biosciences) in serum-free DMEM. Medium containing 10% FBS was placed in the lower chamber as a chemoattractant. After 48 hours, inserts were fixed and stained with crystal violet, non-migrated cells removed using cotton swabs and images captured using an Olympus CKX41 microscope (Olympus, Tokyo, Japan), with a 20X LCAch objective and registered using analysis getIT software (Olympus)

### Clonogenic assay

Single cell suspensions were seeded in 6-well plates at a density of 300 cells per well in 10% FBS DMEM. After 2 weeks, colonies were fixed and stained with crystal violet. Wells were photographed and colonies counted.

### Wound healing assays

Cell migration was assayed using 24-well plates with IBIDI Culture-Inserts (IBIDI GmbH, München, Germany), where no cell damage occurs. 40000 cells were seeded into the two reservoirs of the same culture insert and incubated until confluence was reached. Inserts were removed and migration was monitored by taking pictures every 12 hours using a Leica DM IL microscope, with a 10X Plan Fluotar objective.

### Patients and samples

80 patients as training and 120 as validation set of stage II CRC patients undergoing surgery between 2000–2004 and 2004–2008 respectively in La Paz University Hospital, were enrolled in the study. Formalin-Fixed, Paraffin-Embedded (FFPE) samples were obtained with the patient's authorization and with the approval of the human research Ethics review Committee of La Paz University Hospital (HULP-PI-1452). Inclusion criteria: Age ≥18, completely resected rectal cancer or colon adenocarcinoma located at ≥15 cm of the anal verge as determined by endoscopy or above the peritoneal reflection in the surgical resection, confirmed Stage II AJCC/UICC primary CRC and follow-up of at least 36 months. Exclusion criteria: death within 30 days after surgery, other cancers in previous 5 years and inflammatory bowel disease or specific gene-related cancer.

### Statistical analysis

Significance between groups was determined by *t*-test analyses. Data with *P* < 0.05 were considered statistically significant (*, *P* < 0.05; **, *P* < 0.01; ***, *P* < 0.001). Disease free survival (DFS) was estimated using Kaplan-Meier method. Log-rank test and Univariate Cox regression analysis was performed to test the association between DFS and gene expression. Hazard ratios (HR) and 95% CI were calculated from the Cox regression model, adjusted for potential confounding factors. All reported *p* values were two-sided. Statistical significance was defined as *p* < 0.05. The statistical analyses were performed using the R statistical software version 2.15 (http://www.r-project.org).

## SUPPLEMENTARY FIGURES AND TABLES


