# Supplementary material for: A link between lipid metabolism and epithelial-mesenchymal transition provides a target for colon cancer therapy
Source: Oncotarget. 2015 Oct 5;6(36):38719–36. doi: 10.18632/oncotarget.5340 (PMC4770732; doi:10.18632/oncotarget.5340)
Supplement: Supplementary file 1 [file oncotarget-06-38719-s001.pdf]

SUPPLEMENTARY FIGURES AND TABLES

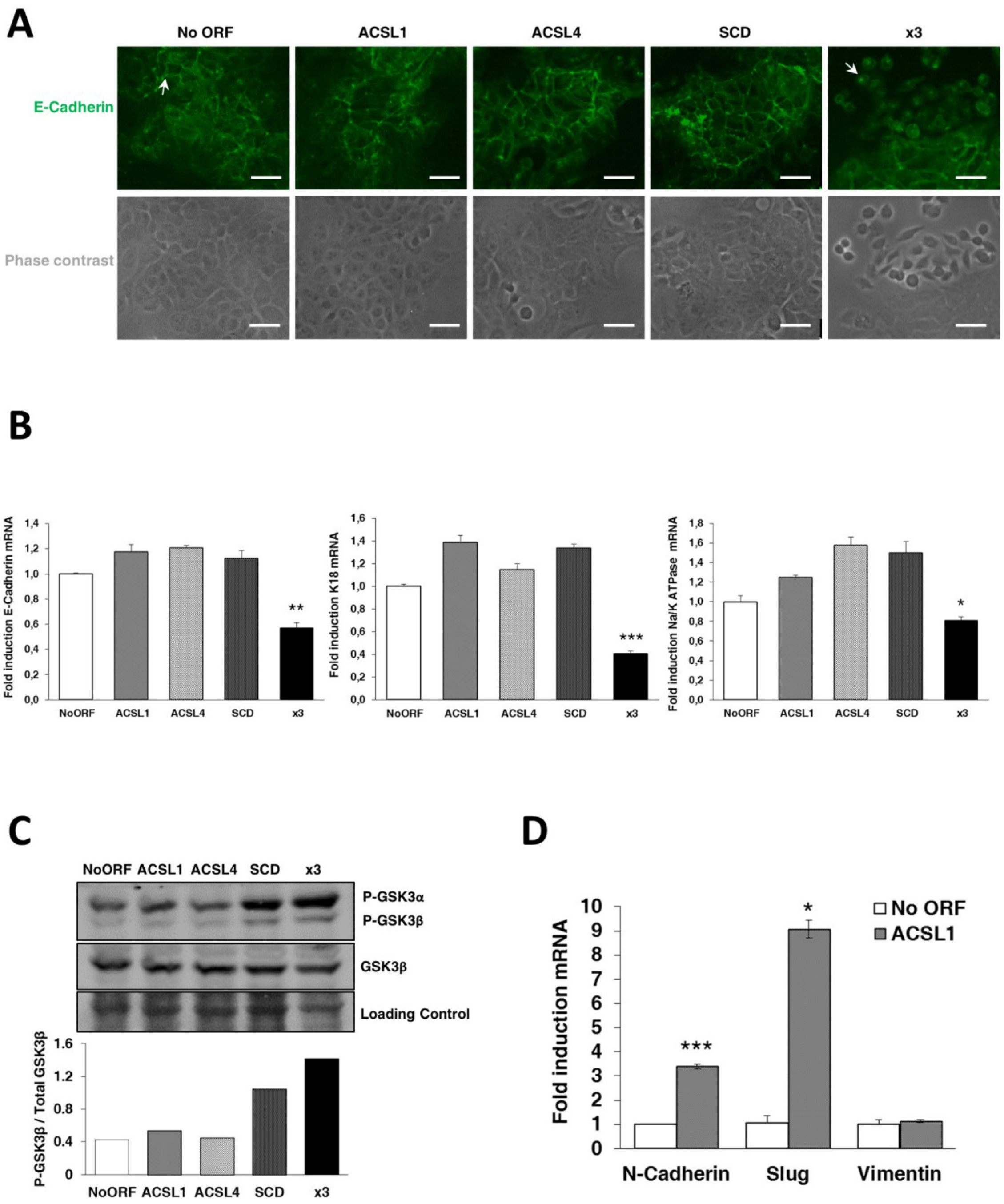

Supplementary Figure S1: Individual overexpression of ACSL1, ACSL4 and SCD is not sufficient to promote complete EMT morphological program. A. Fluorescence microscopy showing normal distribution of E-Cadherin (green) in No ORF, ACSL1, ACSL4, SCD and x3 cells. Arrows indicate the differential cellular distribution of E-Cadherin in control and x3 cells. No morphological changes are observed in phase contrast images (bottom panels) except for x3 cells. Scale bars, 50 µm. **B.** RT-QPCR analysis of epithelial genes *E-Cadherin* (left panel), *Keratin 18* (central panel) an *Na+/K+ATPase β1* (right panel). A decrease in mRNA levels is only found in the case of x3 cells compared to control No ORF and ACSL1, ACSL4 and SCD cells. **C.** Levels of GSK3 phosphorylation detected by Western Blot using a phospho-specific antibody (Ser21/9). Total GSK3β levels detection and Ponceau-stained band served as loading controls. Bottom panel: Quantification of bands intensity showing the ratio of phosphorylated to total protein. **D.** ACSL1 stable overexpression alone increases EMT genes expression levels but this is insufficient to drive a complete phenotypical change. Mesenchymal genes (*N-Cadherin, Slug* and *Vimentin*) expression levels were measured by RT-QPCR. Experiments in B and D were performed in triplicates ($n = 3$). Results represent the mean ±SD ($n = 3$). *, $p < 0.05$, **, $p < 0.01$, ***, $p < 0.001$.

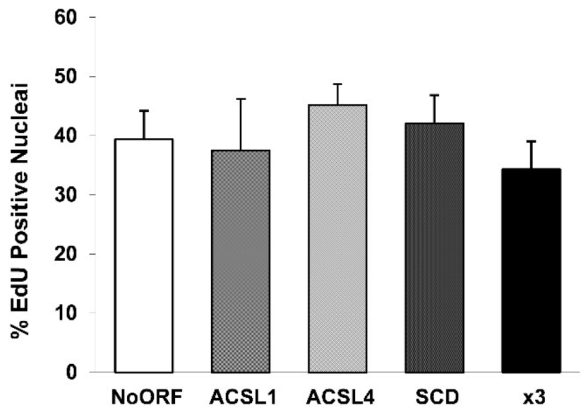

Supplementary Figure S2: Absence of major changes in proliferation upon ACSL1, ACSL4 and SCD overexpression in confluent conditions. Quantification using fluorescence microscopy of EdU incorporation in confluent cells as a measure of cell proliferation. Experiment was performed in triplicates ($n = 3$). Results represent the mean ±SD ($n = 3$).

Supplementary Table S1: Clinical and histopathological characteristics of patients

Characteristics	Stage II CRC					
	Training group			Validation group		
		n° of Patients	(%)		*n*° of Patients	(%)
**Total sample size (*n*)**		77	(100)		119	(100)
**Age at Diagnosis (years)**						
Mean	68.22			66.08		
Median	69			66		
Age Range	32–86			26–91		
<70		42	(54.55)		73	(61.34)
≥70		35	(45.45)		46	(38.66)
**Gender**						
Female		33	(42.86)		54	(45.38)
Male		44	(57.14)		65	(54.62)
**Stage**						
IIA (T3 N0 M0)		56	(72.73)		70	(58.82)
IIB (T4 N0 M0)		21	(27.27)		49	(41.18)
**Total Lymph Nodes Resected**						
Mean Lymph nodes resected	12.09			14.20		
Range of Lymph nodes examined	1–29			0–43		
≤12		46	(59.7)		54	(45.4)
>12		30	-39		62	(52.1)
Unknown		1	(1.3)		3	(2.5)

**Characteristics**	**Stage II CRC**					
	**Training group**			**Validation group**		
		***n*° of Patients**	**(%)**		***n*° of Patients**	**(%)**
**Location of Primary**						
Cecum and Ileocecal Valve		2	(2.6)		13	(10.92)
Acending colon and Hepatic flexure		29	(37.66)		29	(24.37)
Transverse colon		6	(7.79)		6	(5.04)
Splenic flexure and Descending colon		5	(6.49)		17	(14.29)
Sigmoid colon and rectosigmoid junction		34	(44.16)		54	(45.38)
Rectum		1	(1.3)		0	
**Grade/Differentiation**						
Well		5	(6.49)		10	(8.4)
Moderately		66	(85.71)		95	(79.8)
Poor		5	(6.49)		10	(8.4)
Unknown		1	(1.3)		4	(3.4)
**Bowel Obstruction/Perforation**						
Yes		10	(12.99)		45	(37.82)
No		67	(87.01)		74	(62.18)
**Other Histological Features**						
Perineural invasion		12	(15.58)		25	(21)
Vascular invasion		22	(28.57)		31	(26.05)
**Adjuvant treatment**						
Yes		47	(61.04)		76	(63.87)
No		30	(38.96)		43	(36.13)
**Disease-free survival**						
Patients with recurrence		22	(28.57)		18	(15.13)
**Overall survival**						
n° of Exitus		13	(16.88)		11	(9.24)

Supplementary Table S2: Commercial antibodies used in this study

Name	Company	Cat No
Anti-E Cadherin antibody [HECD-1]	Abcam	ab1416
Purified Mouse Anti-β-Catenin	BD Transduction	610154
ACSL1 Rabbit Polyclonal Antibody	Cell Signaling	4047
Phospho-Acetyl-CoA Carboxylase (Ser79) Antibody	Cell Signaling	3661
Acetyl-CoA Carboxylase (C83B10) Rabbit mAb	Cell Signaling	3676
Phospho-Akt (Ser473) Polyclonal Antibody	Cell Signaling	9271
Akt (pan) (C67E7) Rabbit mAb	Cell Signaling	4691
Phospho-GSK-3α/β (Ser21/9) Rabbit Polyclonal Antibody	Cell Signaling	9331
GSK-3β (27C10) Rabbit mAb	Cell Signaling	9315
p-ERK (E4) Mouse mAb	Santa Cruz	sc-7383
ERK2 (C-24) Rabbit Polyclonal Antibody	Santa Cruz	sc-154
Monoclonal Anti-β-Actin, mouse antibody	Sigma	A1978
Horseradish peroxidase conjugated antibody anti-mouse	Millipore	AP308P
Horseradish peroxidase conjugated antibody anti-rabbit	Millipore	AP307P
Alexa 488-conjugated anti-mouse antibody	Invitrogen	A-11001

Supplementary Table S3: Primers used for quantitative real-time PCR

Gene name	forward primer	reverse primer
ACSL1	ACATTATGTTCCTGGGCCCA	AGTCAGAAGGCCATTGTCGA
ACSL4	GGCACAACAGAAAGGGGTAG	GGTTCCTCAGCTCCTTCCTT
SCD-1	TGCCCACCACAAGTTTTCAG	CATCAGCAAGCCAGGTTTGT
E-CADHERIN (CDH1)	GAACGCATTGCCACATACAC	GAATTCGGGCTTGTTGTCAT
KERATIN 18 (KRT18)	GAGTATGAGGCCCTGCTGAA	CAGACACCACTTTGCCATCC
NA+/K+ATPASE B1(ATP1B1)	GCCTCCCAAGAATGAGTCCT	ATTTGGGCTGCAGGAGTTTG
N-CADHERIN (CDH2)	CGGTTTCATTTGAGGGCACA	TTGGAGCCTGAGACACGATT
SLUG (SNAI2)	CGTTTTCCAGACCCTGGTT	CTGCAGATGAGCCCTCAGA
VIMENTIN	GAGTCCACTGAGTACCGGAG	ACGAGCCATTTCCTCCTTCA
COX-2	ATCACAGGCTTCCATTGACC	CAGGATACAGCTCCACAGCA
CD44	AGCAGCGGCTCCTCCAGTGA	CCCACTGGGGTGGAATGTGTCT
LGR5	TCCAACCTCAGCGTCTTCAC	CGCAAGACGTAACTCCTCCA
ALDH1A1	TGTTAGCTGATGCCGACTTG	TTCTTAGCCCGCTCAACACT
EPCAM	CGCAGCTCAGGAAGAATGTG	TGAAGTACACTGGCATTGACG
CD29(ITGB1)	CATCTGCGAGTGTGGTGTCT	GGGGTAATTTGTCCCGACTT
GAPDH	TGGTATCGTGGAAGGACTCATGAC	ATGCCAGTGAGCTTCCCGTTCAGC
